# Proposition of a New POLA Index to Assess the Immunomodulatory Properties of the Diet and Its Relationship with the Gut Microbiota, Using the Example of the Incidence of COVID-19 in a Group of People without Comorbidities

**DOI:** 10.3390/nu14204227

**Published:** 2022-10-11

**Authors:** Paweł Jagielski, Dominika Wnęk, Edyta Łuszczki, Izabela Bolesławska, Agnieszka Micek, Agnieszka Kozioł-Kozakowska, Beata Piórecka, Karolina Koczur, Katarzyna Jankowska, Agata Gaździńska, Marta Turczyńska, Paweł Kawalec

**Affiliations:** 1 Department of Nutrition and Drug Research, Institute of Public Health, Faculty of Health Sciences, Jagiellonian University Medical College,31-066 Krakow, Poland; 2 The Cracow’s Higher School of Health Promotion, 31-158 Krakow, Poland; 3 Institute of Health Sciences, Medical College of Rzeszów University, 35-310 Rzeszów, Poland; 4 Department of Bromatology, Poznan University of Medical Sciences, 3 Rokietnicka Str., 60-806 Poznań, Poland; 5 Department of Nursing Management and Epidemiology Nursing, Jagiellonian University Medical College, 31-007 Cracow, Poland; 6 Department of Pediatrics, Gastroenterology and Nutrition, Institute of Pediatrics, Faculty of Medicine, Jagiellonian University Medical College, 30-663 Kraków, Poland; 7 Department of Endocrinology, Bielanski Hospital, Center of Postgraduate Medical Education, ul. Cegłowska 80, 01-809 Warsaw, Poland; 8 Laboratory of Dietetics and Obesity Treatment, Department of Psychophysiological Measurements and Human Factor Research, Military Institute of Aviation Medicine, Krasińskiego 54/56, 01-755 Warsaw, Poland

**Keywords:** COVID-19 pandemic, dietary intake, dietary inflammatory index, gut microbiota, nutrition, immunomodulation, POLA index

## Abstract

A rise in the incidence of infections with severe acute respiratory syndrome coronavirus 2 has sparked the search for protective strategies against the new pathogen. It is known that individual food components can interact with different immune cells, modulating the immune response of the body. The aim of this study was to develop an index assessing the immunomodulatory potential of diet (POLA index) and to test its utility for the prediction of coronavirus disease 2019 (COVID-19) in a group of healthy young people following a traditional or vegetarian diet. Data on body composition, anthropometric measurements, physical activity, dietary intake, and gut microbiota were obtained from 95 adults (mean age, 34.66 ± 5.76 years). There was a strong correlation between the dietary inflammatory index and the POLA index (r = 0.90; *p* < 0.0001). Based on Cohen’s kappa statistic, there was a good agreement in qualitative interpretation between the two indices (kappa = 0.61; *p* < 0.0001). People on a diet with beneficial immunomodulatory effects had a lower risk of COVID-19 of approximately 80%, as compared with those on a diet with highly unbeneficial immunomodulatory effects. In daily practice, the POLA index might serve as a useful tool for dietitians to identify individuals whose diet is deficient in ingredients for optimal immune system function and change their dietary behavior to ensure optimal immune function that reduces the risk of infection.

## 1. Introduction

The increased incidence of severe acute respiratory syndrome coronavirus 2 (SARS-CoV-2) infections has sparked the search for protective strategies against the new pathogen. In 2021, the theoretical framework started to be established at the Department of Nutrition and Drug Research at the Jagiellonian University Medical College in Kraków, Poland, for developing a simple tool to determine the effect of diet on immunity. Experts from other research centers have been invited to participate in the project. The result of this collaboration is an index that measures the immunomodulatory potential of diet. Its effectiveness was tested by analyzing data on the incidence of coronavirus disease 2019 (COVID-19) and by comparison with the performance of the dietary inflammatory index (DII). The next step will be validation, than a study on a larger group of people, in addition to studies of eating behavior, energy expenditure, body composition and intestinal microbiota; blood samples will be taken for biochemical tests (immunoglobulins, interleukins, vitamin D, etc.) to confirm the link between food components and the gut microbiota and immune system function.

The new index has been developed based on a review of the literature and preliminary results from research [1]. Numerous studies have indicated that immune function is affected by multiple modifiable and nonmodifiable factors. Nonmodifiable factors cannot be changed or controlled and include genetic characteristics, age, and physiological state. On the other hand, modifiable factors can be changed to a varying degree and include healthy diet, physical activity, body weight, sleep duration, and stress [2]. Individual food components can interact with different immune cells, modulating the body’s immune response. In other words, different dietary components are believed to have an immunomodulatory effect. On the other hand, nutritional deficiencies (both quantitative and qualitative) can result in reduced lean body mass, increased oxidative stress, inflammation, and intestinal dysbiosis. All these factors can contribute to a higher susceptibility of the body to viral infections and a more severe clinical course of some diseases. This becomes particularly important in the context of the COVID-19 pandemic and the spread of SARS-CoV-2 [2,3].

Dietary components with immunomodulatory effects include the fat-soluble vitamins A, E and D, water-soluble vitamins such as vitamins C and B, selected minerals (zinc, selenium, iron, magnesium), omega-3 and omega-6 polyunsaturated fatty acids, and polyphenols [4,5]. There is growing evidence that optimizing the diet to include immunomodulatory components may help prevent or alleviate some diseases, including viral respiratory tract infections.

Although the concept of immunomodulation is well known, tools that would help evaluate the impact of current nutrition on immune function are lacking. The only available index is the DII, and it measures the relationship between diet and inflammation [6]. While the DII primarily assesses the anti-inflammatory properties of diet, the POLA index was designed to additionally take into account the numerous biological active compounds that are contained mainly in vegetables, fruits and nuts, and for which there is no data in nutritional databases, while their different mechanisms influence the immune response (such as anti-inflammatory, antiviral, and antibacterial mechanisms, or those stimulating the growth of healthy gut microbiota).

The aim of this study was to develop an index (the so-called POLA index) that would assess the immunomodulatory properties of the diet, and to test its utility for predicting COVID-19 in healthy young people on a traditional or vegetarian diet. Furthermore, we evaluated the relationship between the proposed POLA index and the validated DII.

## 2. Materials and Methods

### 2.1. Subjects

The study group and the methodology used were described in detail previously in the article “*Associations of Nutritional Behavior and Gut Microbiota with the Risk of COVID-19 in Healthy Young Adults in Poland*” [1].

Data from 95 participants were used to calculate the POLA index and DII, and to assess the relationship between these indices and the risk of COVID-19. The mean age of participants was 34.66 ± 5.76 years. None of the participants had obesity or any comorbidities. In addition, they were moderately physically active and were mainly nonsmokers. They followed a traditional or vegetarian diet. Their dietary and lifestyle behaviors were evaluated on the basis of data on eating habits (assessed using a 7-day food diary), physical activity, 24 h energy expenditure (assessed using Polar M430 watches), and body composition (assessed using a Tanita BC-418 MA scale; Tokyo, Japan), as well as intestinal microbiota (assessed by stool culture and polymerase chain reaction in a microbiology laboratory).

### 2.2. Dietary Intake

Participants received detailed instructions on how to prepare the dietary records for 7 consecutive days (both from foods and supplements). Participants were carefully informed to follow their standard diet during the observation week. Then, data were entered into the Dieta 6.0 software (National Food and Nutrition Institute, Warsaw, Poland) in order to calculate the energy and nutritional value of daily food rations. The values of macronutrients and micronutrients were referred to the Polish norm.

### 2.3. DII and the POLA Index

#### 2.3.1. Dietary Inflammatory Index

The DII was calculated based on the method proposed by Shivappa et al. [6]. Briefly, a regionally representative database was used to reflect the diets of various populations and assess the basic statistics for each of the 45 ‘food parameters’, consisting of whole foods, nutrients and other bioactive compounds. In the sample examined, an individual’s food-specific dietary intake was linked to standard global parameters estimated by Shivappa, and the Z scores for each food product were obtained by subtracting the standard mean from the amount of reported consumption and then dividing them by the standard deviation. The conversion to percentile scores and then centralization to zero were applied to improve shape of the distribution. Multiplication by the corresponding overall inflammatory effect score assessed from the global composite dataset allowed us to obtain the specific DII score for food parameters. Finally, the summation of specific DII scores resulted in the creation of the overall DII score. In the original article by Shivappa et al. [6], the 45 food parameters were included in the calculation; however, we had evaluated only 30, namely, onion, garlic, coffee, tea, alcohol, energy, protein, carbohydrates, fiber, vitamins C, D, A, E, B6, B12, B1, B2 and B3, folate, β-carotene, saturated fatty acids, total fat, monounsaturated fatty acids, polyunsaturated fatty acids, omega-3 fatty acids, omega-6 fatty acids, cholesterol, magnesium, iron and zinc. We did not have information about the consumption of eugenol, ginger, saffron, Se, trans fat, turmeric, pepper, thyme/oregano, rosemary and polyphenols (flavan-3-ol, flavones, flavonols, flavonones, anthocyanidins, isoflavones).

#### 2.3.2. POLA Index

##### Theoretical Assumptions

A healthy lifestyle, including a well-balanced diet, moderate physical activity, maintenance of normal body weight, and stress management, contributes to optimal immune function. On the other hand, in addition to rare cases of innate immune dysfunction, lifestyle factors such as dietary errors, smoking, alcohol abuse, obesity, exhaustion, and insufficient sleep can lead to impaired immunity.

The POLA index has been developed based on a similar premise, namely, that good nutrition ensures optimal immune function, while poor eating habits contribute to immune impairment. The index takes into account the supply of minerals, vitamins, and fatty acids that have a proven immunomodulatory effect, including: potassium, magnesium, iron, zinc, calcium, vitamins A, D, E, B1, B6, and C, folate, linoleic acid (18:2), α-linolenic acid (C18:3), and fiber, as well as the intake of fruits, vegetables, and nuts. We realise how important the protein supply is also for the proper functioning of the immune system; however, this component was not included in the POLA indicator, and currently we do not observe protein deficiencies in the population. Moreover, if there is an adequate supply of zinc and iron in the diet, there should also be an adequate supply of protein.

##### Calculation Method

Data on the intake of the ingredients mentioned above were obtained from the 5–7 days (either 7 consecutive days, or 5, i.e., 2 from the weekend and 3 from the week) dietary diary prepared using the current note-taking method, and then entering this information into a computer program for the analysis of the dietary data (Dieta 6.0 Software, National Food and Nutrition Institute, Warsaw, Poland).

It was assumed that if the dietary supply of an ingredient, as an average of the 5–7 days, exceeded 100% of the estimated average requirement (EAR)/recommended dietary allowance (RDA)/adequate intake (AI) standard, a score of 0 points was assigned, and if it was lower than 100%, a score of 1 point was assigned.

Exceptionally, for vitamin D, three categories of supply were arbitrarily adopted: above 100% of the standard, between 50% and 100%, and below 50%, scoring 0, 1, and 2 points, respectively. This classification was justified by the seasonal variability in vitamin D supply (natural sources and supplementation), and the fact that it cannot be produced through skin synthesis.

For fruit and vegetable intake, three categories of intake were adopted: up to 400 g, between 400 and 600 g, and above 600 g, scoring 2, 1, and 0 points, respectively. The score of 2 points for fruit and vegetable intake of less than 400 g per day was adopted to exclude cases when the ingredients were obtained from supplements rather than from food. Another reason was to account for substances with antiviral or antibacterial effects, and polyphenols, that is, bioactive compounds that are found in plant foods but are not included in the database of the food diary analysis software.

Nut consumption of up to 10 g per day and higher than 10 g per day scored 1 and 0 points, respectively.

An overall score of up to 5 points indicates a diet with optimal immune function (beneficial immunomodulation [BIM]); of 6 to 11 points, a diet that slightly weakens the immune function (unbeneficial immunomodulation [UBIM]); and of 12 points or higher, a diet that significantly weakens the immune function (highly unbeneficial immunomodulation [HUBIM]). An example calculation of the POLA index is presented in Table 1.

### 2.4. Statistical Analysis

The following descriptive statistics were calculated: mean, standard deviation, median, and quartile range. Compliance with the normal distribution of quantitative variables was verified using the Shapiro–Wilk test. The analysis of variance (ANOVA) or the Kruskal–Wallis test was used to test for differences between the three groups. The odds ratio and the 95% CI were calculated using multiple logistic regression. Cohen’s kappa statistic was used to assess agreement between the DII and the POLA index, and the results were illustrated using a Sankey diagram. Correlations between quantitative variables were assessed using the Spearman rank correlation coefficient. Statistical analyses were performed with PS IMAGO PRO 7 (IBM SPSS Statistics 27) and STATISTICA 13 software. The level of statistical significance was set at α < 0.05.

### 2.5. Ethics

All participants were informed about the study design and procedures and provided their written consent to participate in the study. The study was carried out in accordance with the Declaration of Helsinki for medical research and was approved by the Jagiellonian University Bioethics Commission (No. 1072.6120.5.2020 and 1072.6120.202.2019).

## 3. Results

### 3.1. Characteristics of Participants

There were no significant differences in age, body mass index (BMI), and physical activity level (PAL) between participants according to the POLA index. Moreover, participants did not differ in the type of diet, marital status, education, and smoking. However, sex differences were found according to the POLA index: among participants on a HUBIM diet, there were significantly more women than among those on a BIM diet. The results are shown in Table 2.

In the study group, 37 participants were on a diet that ensured optimal immune function (BIM), 28 participants were on a diet that slightly weakened the immune function (UBIM), and the remaining 30 participants were on a diet that significantly weakened the immune function (HUBIM).

According to the DII, 32 participants were on an anti-inflammatory diet (tertile 1), and 30 participants were on a pro-inflammatory diet (tertile 3). A total of 33 participants were in the second tertile according to DII.

There were significant differences between the diet groups according to the POLA index in the supply of most dietary components, such as energy, protein, fat, carbohydrates, vitamins, and minerals. No differences were noted for the supply of animal protein, sucrose, and alcohol (Table S1).

In the BIM diet group, dietary intake standards for at least 75% of participants were achieved for most of the individual components, except vitamin D, linoleic acid, and calcium. On the other hand, at least 75% of participants in the HUBIM diet group did not meet the intake standards for linoleic acid, α-linolenic acid, fiber, potassium, calcium, magnesium, zinc, thiamin, folate, and vitamin D (Table 3).

There were significant differences in the intake of selected foods according to the POLA index score, as presented in Table 4. Participants in the BIM diet group showed a significantly higher consumption of seeds, nuts, fruits, vegetables, and legumes than those in the HUBIM diet group. Similar differences between groups were noted for vegetables, fruits, and nuts after these products were divided according to average intake (less than and more than 500 g for vegetables and fruits, and less than and more than 10 g for nuts). Moreover, a separate analysis was performed for onion and garlic according to average intake (less than and more than 1 g per day for garlic, and less than and more than 10 g per day for onions). Again, significant differences in average intake were observed between groups according to the POLA index score, with a higher intake noted for the BIM diet group.

### 3.2. Association of the POLA Index and DII with the Risk of COVID-19

**Figure 1:**
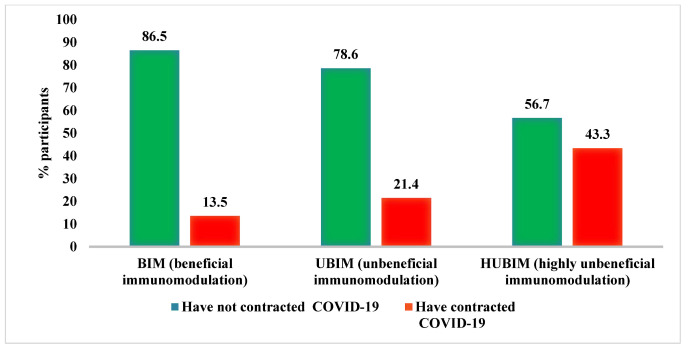
Incidence of COVID-19 according to the POLA index.

The percentage of participants who contracted and did not contract COVID-19 according to the POLA index is shown in Figure 1. In the BIM diet group, only 13.5 % of participants developed COVID-19, compared to 43.3 % in the HUBIM diet group.

**Figure 2:**
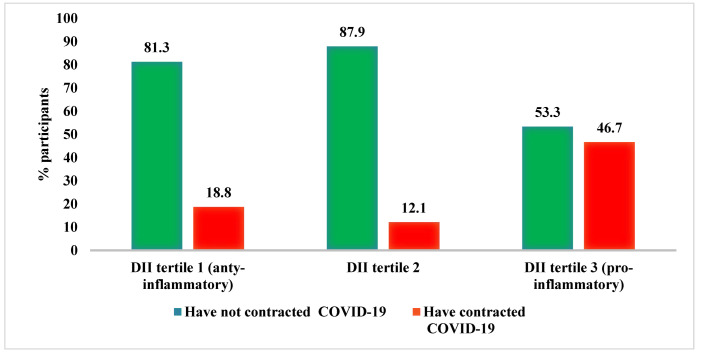
Incidence of COVID-19 in relation to the DII.

The percentage of participants who contracted and did not contract COVID-19 according to DII tertiles is shown in Figure 2.

The risk of COVID-19 according to the categories of the DII and POLA index is shown in Table 5. Participants in the third DII tertile showed almost four-fold greater odds of COVID-19 in a crude model, compared with participants in the lowest DII tertile. However, the difference was no longer significant after adjustment for the type of diet, sex, marital status, age, body fat percentage, smoking, and the presence of *Escherichia coli*. Based on the POLA index, the BIM diet group showed about 80% lower odds of COVID-19 than the HUBIM diet group. The result remained significant regardless of the level of adjustment (OR = 5.29, 95%CI: 1.43–22.55 for HUBIM vs. BIM in the fully adjusted model).

### 3.3. Correlations between DII and POLA Index

**Figure 3:**
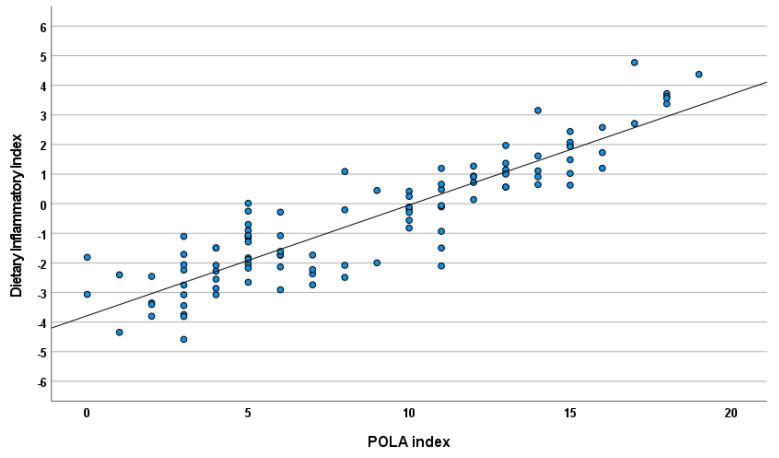
Correlations between the DII and POLA index (r = 0.90; *p* < 0.0001).

**Figure 4:**
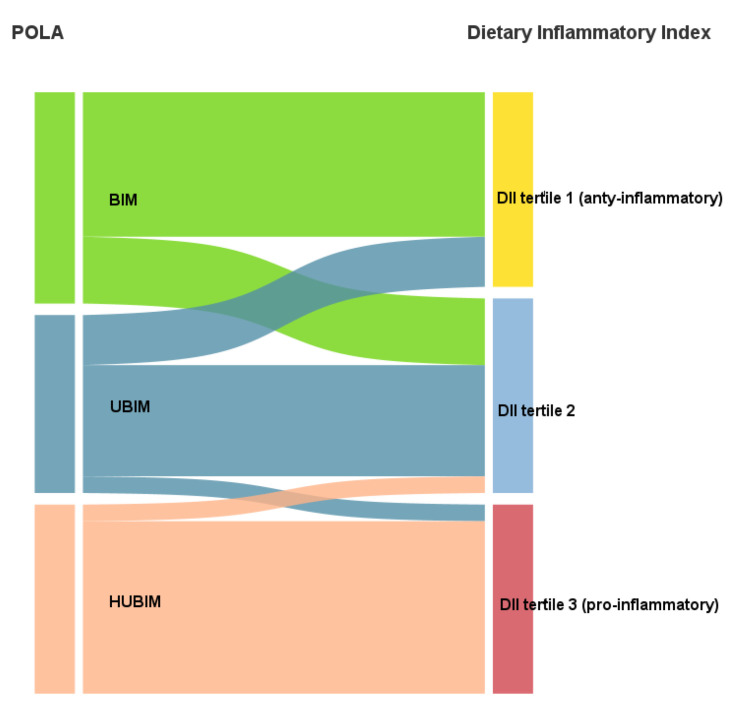
Sankey diagram showing agreement of the DII and POLA index (kappa = 0.61).

There was a strong correlation between DII and the POLA index (r = 0.90; *p* < 0.0001) as presented in Figure 3. Cohen’s kappa test showed good agreement in the qualitative interpretation between the two indices (kappa = 0.61; *p* < 0.0001) (Figure 4).

### 3.4. Gut Microbiota

**Figure 5:**
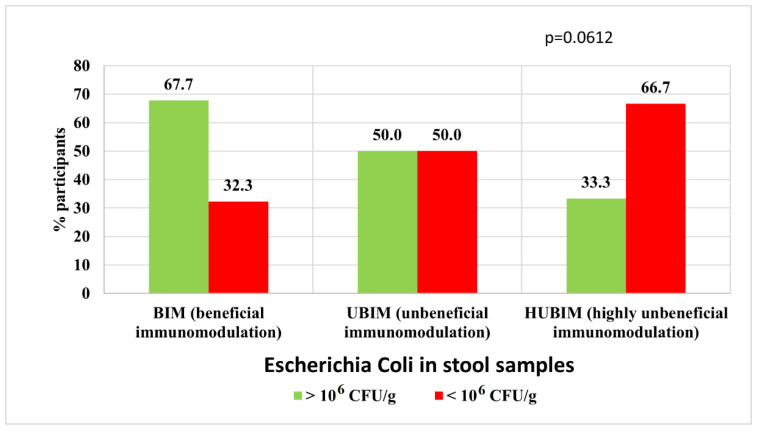
Relationship between an immunomodulatory diet and the amount of *E. coli* (immunostimulatory) among men.

The relationship between the POLA index and the composition and quantity of the immunostimulatory microbiota *E. coli* and *Enterococcus* spp. was analyzed. This relationship was evident in men for *E. coli*, whereas it was not observed for the entire group analyzed. In the BIM diet group, a statistically higher proportion of subjects (67.7%) with normal amounts of *E. coli* bacteria was observed in stool samples (>10^6^ CFU/g) in contrast to the HUBIM group, where only 33.3% had the correct level of this bacterium in the feces (Figure 5).

## 4. Discussion

### 4.1. Diet and the Immune System

The activity of the immune system depends on genetic characteristics, hormones, age, sex, and lifestyle factors such as stress, sleep, and physical activity, but also, to a great extent, on diet. A diet low in micronutrients and macronutrients can weaken the immune system. On the other hand, a well-balanced diet ensures an adequate supply of energy, macronutrients, vitamins, minerals, and many other bioactive ingredients to the body and constitutes an important factor in supporting the immune system.

Proper immune function depends on a close cooperation between two defense mechanisms, namely, nonspecific (or innate) immunity and specific (or acquired) immunity.

Innate immunity is the first line of defense. It is present from birth and comprises passive and active mechanisms. The essential elements of passive defense are the anatomical and physiological barriers: the skin, epithelium, and mucous membranes, which greatly reduce the likelihood that microorganisms will enter the body. If, however, microorganisms overcome these barriers, the mechanisms of innate immunity (complement system, mediating cytokines) are triggered to recognize and eliminate them.

Cells mediating innate immunity show a constant ability to recognize a threat (i.e., it does not change over time) and respond to repeated infections in the same way. They include phagocytic cells (monocytes, macrophages and granulocytes), mast cells, dendritic cells, NK (natural killer) cells, NKT (natural killer T-cells), and ‘innate’ γδ lymphocytes, which are responsible for innate immunity.

The innate response after contact with a pathogen is very rapid and is often sufficient to remove the pathogen from the body.

Acquired immunity is the body’s last line of defense against infection. Most of its mechanisms develop at birth and change over lifetime. Acquired immunity is characterized by a specific memory that comes from a previous contact with a pathogen. Cells involved in acquired immunity, T- and B-lymphocytes, require more time to develop such a high selective recognition of individual infectious agents and to be able to eliminate them effectively. After initial contact with a pathogen, it takes a few days or even weeks for the immune response to develop. However, the next time the immune cells come in contact with this pathogen, they are already able to eliminate it quickly and effectively from the body [7].

In addition to antiviral strategies, the support of immune factors and the modulation of immunosuppressive mechanisms are the primary mechanisms of immunomodulation in the treatment of COVID-19 [8].

In this context, increasing attention is being paid to diet, especially its role in prevention as well as treatment and recovery of patients. Certain dietary macronutrients and micronutrients were found to have immunoregulatory properties from the initial virus–host interaction, through innate immune activation, to adaptive immune response [8,9] and their adequate supply may reduce the risk of the incidence of COVID-19, the severity of symptoms, and the duration of the disease [10,11,12]. On the contrary, the dietary deficiency of numerous macronutrients and micronutrients may increase the risk of COVID-19 or aggravate its course [8].

An effective immune response depends on a tightly regulated balance between proinflammatory and anti-inflammatory mechanisms, involving both the innate and adaptive arms of the immune system [13]. The activation of the immune response induces a broad spectrum of different mediators, such as chemokines, cytokines, pro-oxidant and antioxidant compounds, proinflammatory metabolites, molecules capable of actively counteracting inflammation and promoting its proper resolution, defined specialized pro-resolving mediators and several costimulatory proteins [13]. For an immune response to be effective, substrates necessary to increase the number of immune cells should be available. This includes the synthesis of proteins and complex lipids as well as the availability of substrates that support this, including vitamins and minerals as cofactors [14,15,16,17].

### 4.2. Vitamins, Minerals, and Unsaturated Fatty Acids

Every day, we consume a combination of different foods that interact with each other in complex ways [18]. Most micronutrients show pleiotropic immunostimulatory effects [19]. However, the effect of single nutrients may be insufficient, while the combination of nutrients and foods within a dietary pattern may act synergistically [20] to increase the immunoregulatory potential of the diet. Greene et al. [21] reported a negative correlation between adherence to the Mediterranean diet and the incidence and mortality rates of COVID-19 in selected European countries. In a study by Ponzo et al. [22], participants who reported SARS-CoV-2 infection showed a significantly lower adherence to the Mediterranean diet as assessed by the MeD index than participants without infection. In another study, adherence to standard treatment and the introduction of a hospital diet enriched with vitamins (including B vitamins), minerals, fiber, omega-3 fatty acids, amino acids, and probiotics significantly increased survival and reduced mortality, mechanical ventilation time, and intubation time in patients with stage III COVID-19 [23].

The evaluation of an individual’s diet and nutritional status may be critical to determining comprehensive interventions for the prevention of many diseases, including COVID-19 [8]. Based on this, a novel POLA index was developed, in which the intake of vegetables, fruit, and nuts, as well as the supply of the following 15 food components, was linked to the immunomodulatory effect of diet: potassium, magnesium, iron, zinc, calcium, vitamins A, D, E, B1, B6, and C, folate, linoleic acid (18:2), α-linolenic acid (C18:3), and fiber.

The above components appear to be particularly strongly linked to the immunomodulatory effects of diet. Vitamins A and D and their metabolites are direct regulators of gene expression in immune cells and play a key role in the maturation, differentiation, and response of immune cells [24,25]. Similarly, polyunsaturated fatty acids (PUFA) (essential fatty acids) and fiber are important in maintaining the continuity of mucous membranes that are the first line of defense against numerous pathogens [26,27]. Short-chain fatty acids (SCFAs) produced by the gut microbiota from fiber, are regulators of the immune system [28,29]. In the immune system, calcium signals play a key role in proliferation, differentiation, apoptosis, and transcription of numerous genes [30]. Store-operated calcium channels are also crucial for immune cell activation, T- and B-cell receptor signaling and activation, antigen presentation by dendritic cells, neutrophil and macrophage bactericidal activity, and mast cell degranulation [31]. The development of a pro-oxidant environment through the production of harmful reactive oxygen species (ROS) is one of the components of innate immunity. The host requires protection against reactive oxygen species that is provided by classic antioxidant vitamins (vitamins C [32], E [33], and B-carotenoids [34]) and endogenous antioxidant enzymes (superoxide dismutase, catalase and glutathione peroxidase). The latter require zinc [35], iron [36], and selenium [37].

Additionally, metabolites derived from omega-3 and omega-6 fatty acids have an important function in immune system regulation [9]. Linoleic acid (LA) is a precursor of omega-6 PUFAs in mammals, and its deficiency at the cellular level disrupts the cell–cell interaction by modifying cell–cell adhesion, which can cause abnormal formation of immune synapses, impaired antigen presentation and inadequate lymphocyte activation [38]. LA itself also exhibits anti-inflammatory effects [39]. Importantly, the LA carbon chain can, through elongation and desaturation, be converted to arachidonic acid (AA) and stored in immune cells. Similarly, alpha-linolenic acid (ALA) can serve to synthesize eicosapentaenoic acid (EPA) and docosahexaenoic acid (DHA) [40].

Inflammation is a key component of the protective immune response to noxious stimuli. However, uncontrolled inflammation causes a number of abnormalities in the body [13]. Specialised pro-resolution mediators (SPM)—lipoxins, resolvins, protectins, and maresins derived from essential omega-3 and omega-6 polyunsaturated fatty acids—play an important role in relieving inflammation [41,42]. They limit the further recruitment of neutrophils to the site of inflammation, increase macrophage phagocytic activity, and enhance neutrophil apoptosis. It was suggested that specialized pro-resolving mediators may have a beneficial effect in adjunctive treatment of patients with severe forms of SARS-CoV-2-associated infection to counteract the “cytokine storm” observed in these individuals [13].

Clearly, the 15 dietary components adopted for the purpose of developing the POLA index do not exhaust the list of components with similar proven immunomodulatory benefits, but they appear sufficient to identify individuals with an adequate diet in terms of beneficial immunostimulatory effects and those who require dietary intervention. Participants in the BIM group showed the highest intake levels of the 15 components that determine the immunomodulatory profile of the diet according to the POLA index, as compared with the remaining groups. Importantly, they also showed the highest intake levels of the other components (not included in the POLA index) as well as the highest percentage of compliance with dietary intake standards for individual components. Therefore, we conclude that the analysis of the intake levels of the 15 nutrients included in the POLA index is sufficient to assess the immunomodulatory potential of the diet. The lack of an upper intake limit may raise some concerns, especially for components such as iron, because excess iron promotes the formation of ROS or LA. However, the antioxidant potential of the diet (antioxidant vitamins, polyphenols) seems to prevent the possible adverse effects of excessive amounts of iron and LA.

### 4.3. Vegetables and Fruits

To determine the immunomodulatory potential of diet using the POLA index, we selected three food groups with confirmed effects on the immune system. The beneficial effects of fruit and vegetable consumption on health are due to their content of vitamins A, C, and E, minerals, dietary fiber, and biologically active substances such as polyphenols, carotenoids, and others. These components support the normal function of the immune system by promoting antibody production, lymphocyte proliferation, inhibition of oxidative stress and also by influencing the gut microbiota [11,12].

Epidemiological studies indicated that fruit and vegetable consumption is associated with a lower risk of upper respiratory tract infections such as the common cold, influenza, and sinusitis [43]. Yedjou et al. reported a negative correlation between fruit and vegetable consumption and the incidence and mortality rates of COVID-19 [44]. Bousquet et al. found that the consumption of fermented vegetables or cabbage was associated with low mortality from COVID-19 in European countries, as these products are known to strengthen the immune system of patients with obesity, diabetes, cardiovascular disease, and chronic kidney disease [45].

In the present study, it was observed that individuals on a diet that ensured optimal immune function consumed significantly more vegetables and fruits compared with those who were on a diet that contributed to significant immune impairment. Beneficial diets were also rich in garlic, which contains allicin with anti-inflammatory, antioxidant, and antiviral properties [46]. In addition, they were characterized by high onion consumption, and onions are known to be rich in organosulfur compounds, phenolic compounds, polysaccharides, and saponins, with protective activity against cardiovascular and respiratory disease as well as antioxidant, antimicrobial, anti-inflammatory, antidiabetic, anticancer, and immunomodulatory effects [47]. Vegetables and fruits are also a source of nitric oxide. A high intake of vegetables and fruits rich in nitrates and nitrites (consequently converted to nitric oxide in the body), such as beetroot, leafy vegetables, apples, citrus fruits, and berries, can provide health benefits. Nitric oxide (NO) is formed in the body from the conversion of L-arginine to L-citrulline [48]. Studies also showed that nitric oxide has antiviral effects. It can inactivate virus particles or inhibit virus replication, which has been reported both for DNA and RNA viruses, including coronaviruses. Nitric oxide can also act indirectly by modulating the immune system [49]. Studies revealed that COVID-19-related complications are associated with reduced endothelial nitric oxide production [50,51]. It was also suggested to be an intervention to reduce the risk of SARS-CoV-2 infection, as well as a COVID-19 treatment [52].

### 4.4. Nuts and Pulses

Participants on a diet with positive immunomodulatory effects were also found to have the highest intake of nuts and pulses, which contain high amounts of fiber, resistant starch, arginine, vitamins, minerals, and other bioactive components [53]. Numerous studies confirmed the beneficial effects of walnut, pistachio, or almond consumption on the gut microbiota. Bamberger et al. [54] showed that an 8-week intake of approximately 40 g of walnuts per day resulted in a statistically significant increase in probiotic butyric acid-producing bacteria of *Ruminococcaceae* and *Bifidobacteria*. At the same time, a reduction in pathogenic bacteria of the genus *Clostridium* was observed. An increase in beneficial gut microbiota and a reduction in pathogenic gut microbiota was also reported for pistachio and almond consumption [55]. Finally, observational and experimental studies in adults with and without type 2 diabetes confirmed the beneficial effects of pulse consumption on the lipid profile, blood glucose and blood pressure, which are the main modifiable risk factors for cardiovascular disease [56].

### 4.5. Gut Microbiota

Modification of the intestinal microbiome through an appropriate diet composition can alter the immune response (a key contributor to low-grade inflammation) and metabolic markers. The composition and diversity of the gut microbiota affect the airway microbiota as well as innate and adaptive immunity [8].

In our study, the POLA index showed agreement between the immunomodulatory diet and immunostimulatory bacteria (*E. coli*) in male participants. *E. coli* is a predominantly facultative anaerobic Gram-negative bacterium that colonizes the gastrointestinal tract. *E. coli* strains can act as commensal strains by producing essential vitamins (vitamin K2 and B vitamins), maintaining an anaerobic environment for other gut microbes and excluding pathogenic competitors, but also pathogenic strains of *E. coli* that cause urinary tract infections, bloodstream infections, sepsis and meningitis, among others [57,58].

Commensal E. coli strains produce high concentrations of acetate, formate, propionate, ethanol and lactate [59], as well as short-chain fatty acids [60].

In the human and animal intestines, commensal *E. coli* prevent the accumulation of pathogens, thus protecting against severe intestinal infections [61]. Furthermore, symbiotic *E. coli* strains such as *E. coli* Nissle 1917 are credited with promoting intestinal homeostasis, and flagellin derived from these *E. coli* strains has been proposed as a potential therapy to restore intestinal immune homeostasis [62]. *E. coli* Nissle 1917 can also stimulate the production of human *β*-defensin 2, which can protect the mucosal barrier against adhesion and invasion by pathogenic commensals [63,64]. As commensals, *Enterococci* also colonize the gastrointestinal tract and are involved in modulating the immune system in humans and animals [65].

### 4.6. Nucleotides: A Potential Role in the Development of Immunity

Nucleotides are low-molecular-weight compounds that play a critical role in almost all biochemical processes [66]. 

Nucleotides are not essential components of the diet, but there may be an increased need for them during intensive growth (infants), stress, and physical exertion. Nucleotide deficiency affects cellular and humoral immunity [67].

It is known that dietary nucleotides modulate the immune system, but the underlying molecular mechanisms are still unknown. Initially in animal studies, nucleotides were observed to affect macrophage phagocytosis and lymphocyte subset populations [68]. These results inspired investigators to improve the immunity of newborns, including those that are not breastfed. In these studies, formula milk enriched with nucleotides was shown to have a beneficial effect on the immune system of children by enhancing the maturation, activation, and proliferation of lymphocytes as well as the production of immunoglobulin [69,70].

The benefits of nucleotide supplementation were also observed in control studies in athletes. Exogenous nucleotides may have a protective effect on the immune response markers of athletes after strenuous exercise. Supplementation with nucleotides for 4 weeks counteracted the weakening of the immune system after intense exercise [71].

Previous studies also reported that exogenous nucleotides improved vaccine responses, reduced morbidity, and increased tolerance to antigens [72,73].

Fast-growing tissues, or those that have retained the potential for growth and regeneration, such as meat (muscles), seafood, legumes, and mushrooms, are particularly rich in nucleotides. According to Adjei et al., beef, pork, lamb, poultry, and liver meat, as well as meat and fish extracts (broth), are particularly rich in nucleic acids [74]. Perhaps for this reason, broth has been used for years as a home treatment for colds. However, it should be emphasized that there is a lack of knowledge about the content of nucleotides in consumed food, and the available data are conflicting.

### 4.7. Strengths and Limitations

The POLA index is easy to calculate, accounts for the numerous and diverse mechanisms that affect the immune response (such as anti-inflammatory, antiviral, and antimicrobial mechanisms), and shows a strong correlation with the risk of COVID-19.

The POLA index also shows an immunostimulatory relationship with the gut microbiota in men. While the theoretical assumptions of the POLA indicator are sound, we are aware that testing it on a group of 95 people may be subject to some error, especially as there were few women in the study group. The study of the gut microbiota was not carried out using NGS (next-generation sequencing), but by culture. In addition, the history of COVID-19 was determined based on the declarations of participants, who confirmed the infection using a PCR or antibody test. Blood samples have not been taken and precise biochemical tests of parameters related to the immune system have not been carried out. Currently, the POLA indicator has been tested on a group of people aged 25–45 years, non-obese, without comorbidities, non-smokers, and for such should currently be used until further studies are carried out to confirm its effectiveness for the general population.

Further studies should be carried out with a larger number of people, equally women and men, aged 18–65, both normal weight and obese, so that it can be applied to the general population. Such a study, in addition to an analysis of body composition, gut microbiota, energy expenditure, and eating behaviour, would also include blood sampling and thorough biochemical testing of parameters related to the immune system. Such validation is planned as a further step to confirm the preliminary study results. In Table S2 in the supplementary files, we have shown the rich sources of nutrients included in the POLA indicator, which may be a guide on how to improve the diet of people with HUBIM.

### 4.8. Summary

The beneficial role of diet in terms of ensuring optimal immune function, as determined by the POLA index, is thus far confirmed by our results showing that the group on a diet with beneficial immunomodulatory effects had the highest proportion of participants who did not develop COVID-19. On the other hand, the highest proportion of participants who developed COVID-19 was found in the group on a diet with highly unbeneficial immunomodulatory effects. Similar results were obtained by assessing the inflammatory potential of the diet using the DII. Our study showed a high level of agreement between these indices. For its calculation, 5–7 day dietary diaries (either 7 (including 2 from the weekend), or 5 consecutive days, i.e., 2 from the weekend and 3 from the week) are needed.

Higher POLA index values were associated with significant impairment of immune function and a five-fold higher risk of COVID-19. Similarly, lower POLA index values were associated with a lower risk of COVID-19, indicating optimal immune function. Our findings are in line with the study by Moludi et al., who showed that the risk of COVID-19 was seven-fold higher among participants on a proinflammatory diet versus those on an anti-inflammatory effect, as assessed using the DII [75].

Although the POLA index in this study was assessed in the setting of COVID-19, we assume that it has a much wider application, given that the human immune system is constantly exposed to various pathogens. However, more research is needed to perform biochemical analyses as well as assess the impact on other respiratory infections.

## 5. Conclusions

We propose that the POLA index is used to assess the immunomodulatory potential of a diet, depending on the dietary intake of immune-enhancing nutrients. In daily practice, it might serve as a useful tool for dietitians to identify individuals whose diet is deficient in ingredients for optimal immune system function and guide changes in their dietary behavior, resulting in optimal immune function and a reduced risk of infection. We would like this indicator to be used by researchers investigating the relationship between diet, the immune system and the incidence of, for example, infectious respiratory diseases.

## Figures and Tables

**Table 1:** Example calculation of the POLA index.

	Ingredient	Standard	Dietary Intake in % of Standards or g (the Average of the 5–7 Days)	Points
1	Potassium	AI	10	1
2	Magnesium	RDA	30	1
3	Iron	RDA	78	1
4	Zinc	RDA	120	0
5	Vitamin A	RDA	121	0
6	Vitamin E	AI	15	1
7	Thiamin	RDA	44	1
8	Vitamin B6	RDA	78	1
9	Vitamin C	RDA	123	0
10	Linoleic acid LA	AI	110	0
11	α-Linolenic acid ALA	AI	200	0
12	Fiber	AI	56	1
13	Folates	RDA	78	1
14	Calcium	RDA	34	1
15	Vitamin D	RDA (2 points <50%, 1 point 50-100%, 0 points > 100%)	10	2
16	Vegetables and fruit	(2 points < 400 g, 1 point 400 g–600 g, 0 points > 600 g)	240	2
17	Nuts	1 point < 10 g, 0 points >10 g	5	1
Total score				14
Qualitative interpretation		Diets that significantly weaken the immune function		

RDA—recommended dietary allowance, AI—adequate intake.

**Table 2:** Characteristics of subjects according to POLA index scores.

Variable		BIM N = 37		UBIM N = 28		HUBIM N = 30		ANOVA
		X ± SD		X ± SD		X ± SD		*p*
Age [years]		34.2 ± 6.2		34.1 ± 5.2		35.7 ± 5.8		0.4726
Body weight [kg]		76.1 ± 12.1		73.2 ± 11.0		70.3 ± 13.2		0.1563
Height [cm]		179.8 ± 8.3		177 ± 8.6		173.5 ± 9		0.0130
BMI [kg/m2]		23.5 ± 3.1		23.3 ± 2.5		23.2 ± 2.7		0.9136
TEE [kcal]		2668 ± 445		2601 ± 366		2347 ± 456		0.0089
PAL		1.52 ± 0.2		1.52 ± 0.17		1.45 ± 0.11		0.1470
Sleep duration [h]		7:34 ± 0.53		07:03 ± 00:44		07:24 ± 00:44		0.0428
Steps		12,103 ± 4979		12,300 ± 5087		11,846 ± 3382		0.9305
DII		−2.26 ± 1.12		−0.81 ± 1.18		1.69 ± 1.13		<0.0001
POLA		3.57 ± 1.42		9.04 ± 1.84		14.47 ± 1.89		<0.0001
**Variable**	**Category**	**N**	**%**	**N**	**%**	**N**	**%**	**Chi^2^** ***p***
Sex	Men	31	83.8	24	85.7	18	60.0	0.0299
	Women	6	16.2	4	14.3	12	40.0	
Diet	Traditional	18	48.6	17	60.7	19	63.3	0.4276
	Vegetarian	19	51.4	11	39.3	11	36.7	
BMI [kg/m^2^]	Normal body weight	21	56.8	21	75.0	22	73.3	0.2099
	Overweight	16	43.2	7	25.0	8	26.7	
Marital status	Single/divorced	21	56.8	15	53.6	12	40.0	0.3664
	Married/cohabiting	16	43.2	13	46.4	18	60.0	
BF [%]	Underfat	5	13.5	3	11.1	1	3.3	0.6582
	Normal	27	73.0	19	70.4	23	76.7	
	Overfat	5	13.5	5	18.5	6	20.0	
Smoking	No	34	91.9	25	89.3	24	80.0	0.3238
	Yes	3	8.1	3	10.7	6	20.0	
Education	Secondary	5	13.5	2	7.1	0	0.0	0.2364
	Higher	32	86.5	26	92.9	30	100.0	
How do you rate your physical activity in your free time?	Low	8	21.6	4	14.3	7	23.3	0.2606
	Moderate	16	43.2	17	60.7	19	63.3	
	High	13	35.1	7	25.0	4	13.3	
Vitamin supplementation	I do not use	9	24.3	9	32.1	12	40.0	0.4853
	Periodically	9	24.3	8	28.6	4	13.3	
	Regular	19	51.4	11	39.3	14	46.7	
Minerals supplementation	I do not use	19	54.3	15	57.7	17	56.7	0.9860
	Periodically	10	28.6	6	23.1	7	23.3	
	Regular	6	17.1	5	19.2	6	20.0	

TEE—total energy expenditure, BMI—body mass index, BF—body fat, PAL—physical activity level, N—number of participants, X—arithmetic mean, SD—standard deviation, BIM—beneficial immunomodulation, UBIM—unbeneficial immunomodulation, HUBIM—highly unbeneficial immunomodulation. Bold values denote statistical significance at the *p* < 0.05 level.

**Table 3:** Percentage of achieving dietary intake standards for macronutrients, individual vitamins, and minerals from consumed food and supplements.

Variable	BIM N = 37	UBIM N = 28	HUBIM N = 30	Kruskal–Wallis Test *p*
	Me (Q1–Q3)	Me (Q1–Q3)	Me (Q1–Q3)	
Water	127 (100–154.8)	109.7 (91.3–127.2)	83.7 (73.6–95.1)	**<0.0001**
Total protein [g]	134 (120.2–164.9)	117.8 (100.2–154)	105.1 (97.3–119.9)	**<0.0001**
Fat [g]	84.6 (71.9–105.4)	70.7 (63.5–84.8)	60.8 (55.5–76.4)	**0.0001**
Fats: total saturated	153.8 (111.3–184.7)	137.7 (101.7–171.1)	133.6 (94.3–155.5)	0.2594
Linoleic acid LA (C18:2)	103.9 (86.5–146.5)	73.5 (60.7–88.4)	59.7 (47.8–72.3)	**<0.0001**
α-Linolenic acid ALA (C18:3)	156.8 (116–218.4)	103.9 (78.1–151.7)	80.3 (70.6–91.9)	**<0.0001**
Assimilable carbohydrates	228.2 (200.8–264.9)	204.3 (173.5–225.9)	177.4 (152.8–212.3)	**<0.0001**
Dietary fiber	150.3 (123.2–184.1)	95.3 (82.2–113.4)	70.6 (63.4–83.8)	**<0.0001**
Potassium [mg]	128.4 (120.8–146.4)	100.6 (91.8–108.6)	76.2 (67.9–89.2)	**<0.0001**
Calcium [mg]	98.2 (81.7–111.3)	80.3 (65–105.1)	75.4 (62.6–86.3)	**0.0027**
Magnesium [mg]	142.1 (119.4–162.4)	99.6 (89.7–113.7)	83.1 (72.8–93.4)	**<0.0001**
Iron [mg]	186 (146.7–226.3)	154.9 (123.7–170.3)	97.3 (62.9–125.2)	**<0.0001**
Zinc [mg]	138.9 (124.1–163)	109.8 (92.2–127.2)	92.7 (85.7–98.8)	**<0.0001**
Copper [mg]	263.3 (209.5–307.6)	181.5 (153.1–207.1)	134.6 (115.3–158.3)	**<0.0001**
Manganese [mg]	405.8 (309.9–572.6)	263.7 (207.8–370.1)	200 (162.8–250.8)	**<0.0001**
Vitamin A [µg]	168.2 (129.7–214.3)	145.5 (90.8–172.2)	103.4 (83.1–133)	**0.0001**
Vitamin E (alpha-tocopherol equivalent) [mg]	172 (145.3–202.4)	111.3 (98.1–143.3)	90.6 (70.6–115)	**<0.0001**
Thiamin [mg]	142.2 (122.8–162.7)	101.8 (90.4–111.3)	76.5 (68.9–92.8)	**<0.0001**
Riboflavin [mg]	163.2 (135.9–201.7)	137.8 (107.2–161.1)	120.4 (103–132.4)	**<0.0001**
Niacin [mg]	139.2 (109.7–178.5)	131.3 (102.4–162.9)	101.2 (75.7–117.2)	**0.0003**
Vitamin B6 [mg]	196.8 (174.1–242)	149.3 (130.8–188.5)	110 (101.3–123.6)	**<0.0001**
Folates [mg]	120.6 (101.8–139.4)	93.5 (72.9–106)	64.6 (48–78.2)	**<0.0001**
Vitamin B12 [µg]	188.4 (125.6–375.1)	150.3 (126.1–220.1)	116.4 (106.2–147.9)	**0.0094**
Vitamin C [mg]	179.2 (129.3–243.4)	133.6 (87.5–160.3)	73.2 (59.5–117.6)	**<0.0001**
Vitamin D [µg]	24.4 (18.5–125.9)	25.6 (11.7–64.9)	15.8 (10.5–36.1)	0.0501

N—number of participants, Me—median, Q1 and Q3—lower and upper quartile, BIM—beneficial immunomodulation, UBIM—unbeneficial immunomodulation, HUBIM—highly unbeneficial immunomodulation, bold values denote statistical significance at the *p* < 0.05 level.

**Table 4:** Consumption of food products in dietary groups divided according to the POLA index.

Variable		BIM N = 37		UBIM N = 28		HUBIM N = 30		Kruskal–Wallis Test *p*
		Me (Q1–Q3)		Me (Q1–Q3)		Me (Q1–Q3)		
Groats and rice [g]		20.7 (6.1–46.5)		18 (9.3–32.8)		12.1 (0–20.6)		**0.0433**
Seeds [g]		4.6 (2–12.5)		0.4 (0–3.2)		1.2 (0–3.7)		**0.0002**
Nuts [g]		25 (13.7–43.1)		11.4 (2–20.9)		3.1 (0.6–14.1)		**<0.0001**
Seeds and nuts [g]		37.3 (22.8–60.6)		13.2 (3.3–26.5)		6.7 (1.4–17.2)		**<0.0001**
Fruits [g]		334.7 (244.1–478.9)		151.3 (63.8–257.2)		148.3 (110.1–205.2)		**<0.0001**
Vegetables [g]		443.6 (361–555.5)		341.3 (237.6–478.6)		224 (148.4–281.1)		**<0.0001**
Vegetables—other [g]		152 (116.6–192.7)		91.8 (80.6–159.3)		70.7 (49–105.3)		**<0.0001**
Vegetables rich in beta carotene [g]		102 (70.2–136.8)		90.8 (51.6–126.9)		56 (40.5–80)		**0.0017**
Vegetables rich in vitamin C [g]		164.5 (138.7–217.6)		128.2 (81.9–188.5)		64 (36.4–110.5)		**<0.0001**
Total vegetables and fruits (in market products) [g]		823 (638–1003)		530 (444–644)		378 (276–512)		**<0.0001**
Legumes [g]		32.2 (5–109.5)		11 (0–46.6)		2.4 (0–19.4)		**0.0039**
**Variable**	**Category**	**N**	**%**	**N**	**%**	**N**	**%**	**Chi^2^** ***p***
Garlic	<1 g on average daily	27	73.0	22	78.6	29	96.7	**0.0357**
	≥1 g on average daily	10	27.0	6	21.4	1	3.3	
Onion	<10 g on average daily	22	59.5	19	67.9	29	96.7	**0.0019**
	≥10 g on average daily	15	40.5	9	32.1	1	3.3	
Fruits and vegatables	500 g and more per day	34	91.9	16	57.1	9	30.0	**<0.0001**
	Less than 500 g per day	3	8.1	12	42.9	21	70.0	
Nuts	10 g and more per day	31	83.8	15	53.6	10	33.3	**0.0001**
	Up to 10 g per day	6	16.2	13	46.4	20	66.7	

N—number of participants, Me—median, Q1 and Q3—lower and upper quartile, BIM—beneficial immunomodulation, UBIM—unbeneficial immunomodulation, HUBIM—highly unbeneficial immunomodulation, bold values denote statistical significance at the *p* < 0.05 level.

**Table 5:** Relationship between the risk of COVID-19 and the tertiles of DII and POLA index.

Model			
**DII index**	**First tertile**	**Second tertile**	**Third tertile**
**Model 1 ^a^**	1 (ref.)	0.60 (0.14–2.32)	3.79 (1.26–12.63)
**Model 2 ^b^**	1 (ref.)	0.54 (0.11–2.30)	3.39 (0.93–13.83)
**Model 3 ^c^**	1 (ref.)	0.56 (0.12–2.43)	3.51 (0.93–15.18)
**POLA index**	**BIM**	**UBIM**	**HUBIM**
**Model 1 ^a^**	1 (ref.)	1.75 (0.47–6.75)	4.89 (1.57–17.45)
**Model 2 ^b^**	1 (ref.)	1.96 (0.51–7.93)	4.90 (1.37–20.09)
**Model 3 ^c^**	1 (ref.)	2.04 (0.52–8.33)	5.29 (1.43–22.55)

a—crude model, b—adjusted for the type of diet, sex, marital status, age, body fat percentage and smoking, c adjusted for covariates included in b and additionally for *E. coli*, BIM—beneficial immunomodulation, UBIM—unbeneficial immunomodulation, HUBIM—highly unbeneficial immunomodulation.

## Data Availability

The data presented in this study are not publicly available due to confidentiality reasons. These data are available on request from the corresponding author.

## Supplementary Materials

The following supporting information can be downloaded at: www.mdpi.com/article/10.3390/nu14204227/s1, Table S1: Comparison of dietary intake and supplements in dietary groups divided according to the POLA index. Table S2: Sources of nutrients included in the POLA indicator.
