# Supplementary material for: Proposition of a New POLA Index to Assess the Immunomodulatory Properties of the Diet and Its Relationship with the Gut Microbiota, Using the Example of the Incidence of COVID-19 in a Group of People without Comorbidities
Source: Nutrients. 2022 Oct 11;14(20):4227. doi: 10.3390/nu14204227 (PMC9607188; doi:10.3390/nu14204227)
Supplement: Supplementary file 1 [file nutrients-14-04227-s001.zip › Tables S1 and S2.pdf]

Table S1. Comparison of dietary intake and supplements in dietary groups divided according to the POLA index.

Variable	BIM N=37	UBIM N=28	HUBIM N=30	Kruskal–Wallis test
	**Me (Q1 – Q3)**	**Me (Q1 – Q3)**	**Me (Q1 – Q3)**	**p**
Energy [kcal]	2509 (2225 - 2803)	2186 (1903 - 2430)	1826 (1578 - 2042)	**<0.0001**
Water [ml]	2987 (2500 - 3431)	2696 (2282 - 3116)	2014 (1615 - 2379)	**<0.0001**
Total protein [g]	99 (84.1 - 111.3)	87.1 (62.7 - 109.4)	71 (59.7 - 78.5)	**<0.0001**
Animal protein [g]	46 (20.2 - 68.5)	53.3 (17.8 - 70)	40.3 (29.7 - 49.2)	0.5129
Plant protein [g]	50.9 (42.3 - 65.4)	38 (31.2 - 44)	29.7 (23.3 - 33.4)	**<0.0001**
Arginine [mg]	5523 (5031 - 6616)	4314 (3301 - 5280)	3292 (2673 - 3726)	**<0.0001**
Fat [g]	85 (64.9 - 96.3)	71.5 (56.3 - 80.8)	56.9 (45.4 - 69.9)	**<0.0001**
Linoleic acid LA (C18:2) [g]	14 (11 - 16.3)	9.1 (7.6 - 11.5)	7.2 (5.6 - 8.4)	**<0.0001**
α-Linolenic acid ALA (C18:3) [g]	2.4 (1.7 - 3.5)	1.7 (1.4 - 2.3)	1.1 (1.1 - 1.4)	**<0.0001**
Omega–3 fatty acids [g]	2.3 (1.8 - 3.1)	1.6 (1.1 - 2.5)	1.1 (0.9 - 1.3)	**<0.0001**
Omega–6 fatty acids [g]	11.6 (9.6 - 14.3)	7.6 (6.3 - 9.4)	6.3 (4.4 - 6.7)	**<0.0001**
Total carbohydrates [g]	335.7 (301.6 - 390.4)	291.1 (251.2 - 320.4)	252.2 (215.4 - 296.3)	**<0.0001**
Saccharose [g]	44.6 (35.8 - 59.5)	32.7 (25.9 - 50.3)	37.2 (26.5 - 55.9)	0.0936
Dietary fiber [g]	37.6 (30.8 - 46)	23.8 (20.5 - 28.4)	17.7 (15.9 - 20.9)	**<0.0001**
Alcohol [g]	5.1 (0 - 11.5)	7.9 (0.6 - 18.8)	5.3 (0 - 11.3)	0.3642
Potassium [mg]	4493 (4227 - 5123)	3521 (3213 - 3799)	2668 (2376 - 3120)	**<0.0001**
Calcium [mg]	982 (817 - 1113)	803 (650 - 1050)	754 (626 - 863)	**0.0027**
Magnesium [mg]	569 (485 - 635)	409 (349 - 444)	316 (283 - 355)	**<0.0001**
Iron [mg]	20.4 (17.8 - 22.6)	15.5 (12.7 - 17)	11.1 (9.6 - 12.9)	**<0.0001**
Zinc [mg]	14.5 (13.1 - 17.3)	11.2 (9.9 - 13.5)	8.8 (7.9 - 10)	**<0.0001**
Copper [mg]	2.4 (1.9 - 2.8)	1.6 (1.4 - 1.9)	1.2 (1 - 1.4)	**<0.0001**
Manganese [mg]	9 (7.1 - 11.5)	6.1 (4.6 - 7.9)	4.3 (3.5 - 5.3)	**<0.0001**
Vitamin A [μg]	1495 (1167 - 1827)	1243 (817 - 1532)	872 (646 - 1070)	**<0.0001**
Beta-carotene [μg]	5044 (3609 - 9030)	5329 (3352 - 7235)	2960 (2027 - 3809)	**<0.0001**
Vitamin E (alpha-tocopherol equivalent) [mg]	16.2 (14.3 - 18.5)	11 (9.8 - 13.2)	8.8 (6.9 - 10.2)	**<0.0001**
Thiamin [mg]	1.8 (1.6 - 2.1)	1.3 (1.2 - 1.4)	1 (0.9 - 1.2)	**<0.0001**
Riboflavin [mg]	2 (1.8 - 2.4)	1.8 (1.4 - 2.1)	1.5 (1.3 - 1.7)	**<0.0001**
Niacin [mg]	22.2 (17.3 - 27.9)	20.2 (15.7 - 26.1)	15.2 (12.1 - 17.6)	**0.0002**
Vitamin B6 [mg]	2.6 (2.3 - 3.1)	1.9 (1.7 - 2.5)	1.4 (1.3 - 1.6)	**<0.0001**
Folates [mg]	482.5 (407.3 - 557.6)	373.8 (291.6 - 423.8)	258.4 (191.9 - 312.7)	**<0.0001**
Vitamin B12 [μg]	4.5 (3 - 9)	3.6 (3 - 5.3)	2.8 (2.5 - 3.6)	**0.0094**
Vitamin C [mg]	158.9 (116.4 - 203.5)	114.9 (78.7 - 144.3)	62 (46.9 - 105.8)	**<0.0001**
Vitamin D [μg]	3.7 (2.8 - 18.9)	3.8 (1.8 - 9.7)	2.4 (1.6 - 5.4)	**0.0500**

N – number of participants, Me - median, Q1 and Q3 – lower and upper quartile, BIM - beneficial immunomodulation, UBIM - unbeneficial immunomodulation, HUBIM - highly unbeneficial immunomodulation, bold values denote statistical significance at the $p < 0.05$ level.

Table S2. Sources of nutrients included in the POLA indicator.

No.	Nutrient	Rich food sources
1	Potassium	pulses (beans, peas, soya, lentils), vegetables - spinach, broccoli, carrots, potatoes, beetroot, tomatoes; fruit - dried fruit, apricots, bananas, cherries, peaches, strawberries, oranges, plums; seeds and seeds, and milk and dairy products.
2	Magnesium	Nuts (hazelnuts, almonds, pistachios, cocoa), seeds and kernels (sunflower, pumpkin), whole-grain cereal products (brown rice, buckwheat and barley groats, oat and corn flakes, rye and graham bread), vegetables and legumes - broccoli, carrots, parsley, white and green beans.
3	Iron	Iron of animal origin: meat and offal, fish and seafood , eggs,<br>Vegetable iron: pulses (beans, peas, lentils), whole-grain cereals, nuts and seeds, green vegetables (kale, cabbage, lettuce, spinach, parsley).
4	Zinc	Meat and offal (liver), fish and seafood, seeds and seeds (sunflower, pumpkin) whole grain cereal products (buckwheat, brown rice), rennet cheeses (yellow cheese), eggs.
5	Vitamin A	Sources of Retinol - cod liver oil, fatty marine fish (herring, tuna, mackerel), offal (liver), butter, milk and milk products (cream, fatty cottage cheese), eggs<br>Sources of Beta-Carotene - carrots, pumpkin and corn, peppers, green vegetables (kale, cabbage, lettuce, spinach, broccoli, chard), red and orange fruits (dried and fresh apricots, dried and fresh plums, peaches, mangoes, papaya, red grapefruit, currants, raspberries, tomatoes).
6	Vitamin E	Vegetable oils, i.e. rapeseed, sunflower and soybean oils, seeds and seeds (sunflower, pumpkin, grape, sesame), walnuts and almonds, cereal sprouts, green leafy vegetables, oily sea fish, olive oil.
7	Thiamine	Whole grain cereal products, eggs, meat and meat products, seeds and nuts.
8	Vitamin B6	Whole grain cereal products (buckwheat and millet groats, bran and wheat germ), seeds and nuts (sunflower, sesame and walnuts), pulses (lentils, chickpeas, peas, beans), broccoli, potatoes, fish and poultry.
9	Vitamin C	Berries (black and white currants, strawberries, cranberries, blueberries), citrus fruits (lemons, oranges), peppers, brassica vegetables, pickles, broccoli, cauliflower, red peppers.
10	Linoleic acid (LA)	Vegetable oils (e.g. safflower, evening primrose, hemp, grape seed, walnut, sunflower, corn and soya, sesame), seeds and nuts (walnut, pecan, almond).
11	Alpha-linoleic acid (ALA)	Vegetable oils (e.g. flax, rapeseed, soya), nuts and oilseeds (walnuts, linseed and chia seeds), oat and wheat germ.
12	Dietary fibre	Vegetables, fruit, whole grain cereal products, pulses, potatoes.
13	Folate	Green vegetables, mainly leafy vegetables (spinach, broccoli, savoy cabbage), fruit (oranges, avocado, kiwi, mango), pulses, meat and offal (liver), whole grain cereal products and yeast.
14	Calcium	Milk and its products, fish, nuts, pulses (beans, soya), mainly fermented (e.g. tofu), brassica vegetables.
15	Vitamin D3	Cod liver oil, oily marine fish, butter, fatty milk and milk products.
